# Portal Steal During Liver Transplantation in a 31-Year-Old: When Absent Varices Signal Extensive Splenorenal Shunting

**DOI:** 10.7759/cureus.93425

**Published:** 2025-09-28

**Authors:** Noor Abu Zar, Chia Chey

**Affiliations:** 1 Gastroenterology, Queen Elizabeth Hospital, London, GBR

**Keywords:** aclf, acute-on-chronic liver failure, cirrhosis, liver transplantation, portal decompression, portal hypertension, portal steal, portosystemic shunt, splenorenal shunt, variceal bleeding

## Abstract

Extensive portosystemic shunting in portal hypertension creates a paradox: effective decompression protects against variceal bleeding but complicates liver transplantation through portal steal.

We report a 31-year-old man with cryptogenic acute-on-chronic liver failure grade 3 (Model for End-Stage Liver Disease-Sodium (MELD-Na) 31, United Kingdom Model for End-Stage Liver Disease (UKELD) 63). Despite severe portal hypertension on Doppler ultrasound, endoscopy revealed complete absence of oesophageal and gastric varices. CT explained this paradox, demonstrating a large tortuous splenorenal shunt providing highly effective decompression. At transplantation, this protective adaptation caused portal steal, with immediate right posterior sector graft demarcation. Restoration of perfusion required emergent left renal vein ligation.

This case highlights how extensive splenorenal shunting can fully mask varices yet create critical intraoperative challenges. The absence of varices in established portal hypertension should prompt investigation for alternative drainage pathways. Even in young patients, comprehensive vascular assessment is essential, as protective anatomical adaptations can precipitate life-threatening operative emergencies requiring specialized surgical intervention.

## Introduction

Portal hypertension drives the development of portosystemic collaterals as a natural compensatory mechanism to decompress the portal system [1]. In a recent study on patients with cirrhosis, splenorenal shunts had been reported in approximately 27.2% of the cases [2]. While gastroesophageal varices represent the most clinically recognized and feared manifestation developing in approximately 50-60% of patients with liver cirrhosis [3], alternative collateral pathways can negate this by providing effective decompression, fundamentally altering the clinical presentation and surgical complexity. 

Splenorenal shunting represents a distinct form of portosystemic decompression where the splenic vein establishes direct communication with the left renal vein, creating a large-calibre, low-resistance pathway that bypasses the portal system entirely [4]. Unlike gastroesophageal varices that provide incomplete decompression, extensive splenorenal shunts can achieve near-complete portal decompression, effectively eliminating variceal formation and associated bleeding risk. The development of such extensive shunting at a young age is particularly uncommon, as most reported cases occur in older patients with longstanding portal hypertension. This creates a paradoxical clinical scenario where the absence of varices in established severe portal hypertension should prompt comprehensive cross-sectional imaging assessment rather than clinical reassurance, as current endoscopic grading systems provide limited guidance for quantifying portal pressure severity when varices are absent [5].

However, this protective anatomical adaptation creates a surgical challenge during liver transplantation: the same large-calibre shunts that prevent variceal haemorrhage can cause portal steal phenomenon, diverting critical portal flow away from the transplanted liver and threatening immediate graft viability [6]. The hemodynamic significance of these shunts often remains unrecognized until intraoperative visual assessment reveals hepatic sector demarcation, demanding immediate surgical intervention. 

This case demonstrates how protective anatomical adaptations can become operative emergencies, illustrating the critical importance of recognizing that absent varices in established portal hypertension represents a red flag requiring comprehensive preoperative planning for extensive alternative drainage pathways that will complicate transplant surgery. 

## Case presentation

Patient history and physical examination

A 31-year-old gentleman presented with a 12-day history of progressive epigastric pain radiating to the right hypochondrium, exacerbated by movement. He developed fever six days prior to admission, associated with significantly reduced appetite and persistent hiccups lasting 12 days. He reported a one-month history of bilateral lower limb swelling and jaundice of unknown onset. He had experienced a productive cough with white sputum, treated with doxycycline by his general practitioner with improvement. He denied nausea, vomiting, chest pain, dyspnea, or urinary symptoms. Recent bowel habits included constipation, relieved with senna. Past medical history included childhood asthma, currently not requiring treatment. He denied alcohol consumption, recreational drug use, herbal supplements, or known hepatotoxic exposures. His body mass index was 43.3 kg/m². Initial vital signs were a pulse of 101 bpm, blood pressure 174/84 mmHg, temperature 37.3°C, oxygen saturation of 98% on room air, and a respiratory rate of 18 breaths/min. He also had severe hypoglycaemia (2.7 mmol/L), consistent with severe hepatic synthetic dysfunction. Initial assessment revealed profound icterus, bilateral pitting oedema, but no pallor, cyanosis, clubbing, or lymphadenopathy. Cardiovascular examination revealed normal S1 and S2 heart sounds with an audible systolic murmur. Respiratory examination showed clear lung fields with bilateral equal air entry. Abdominal examination demonstrated mild distension with soft, non-tender abdomen, positive bowel sounds, negative Murphy's sign, and visible peau d'orange sign on the lower abdominal wall. Neurological assessment was unremarkable without evidence of hepatic encephalopathy.

Laboratory and etiological assessment

Initial laboratory assessment revealed multi-organ dysfunction involving the liver, kidneys, and coagulation system, consistent with acute-on-chronic liver failure (ACLF). Liver function tests indicated significant hepatic injury and impaired synthetic function, while coagulation studies suggested pronounced coagulopathy. Renal indices were abnormal, reflecting reduced kidney function, and hematologic parameters demonstrated anaemia, thrombocytopenia, and leucocytosis. Severity scoring confirmed ACLF grade 3, with high Model for End-Stage Liver Disease-Sodium (MELD-Na) and United Kingdom Model for End-Stage Liver Disease (UKELD) scores, indicating substantial perioperative risk (see Table 1 for full laboratory details and reference ranges).

**Table 1:** Initial laboratory findings on admission This table presents a summary of the patient's key laboratory parameters on admission (Day 1). Normal reference ranges are provided for interpretation. WBC: White Blood Cell​​​​​​​; INR: International Normalized Ratio; ALP: Alkaline Phosphatase; ALT: Alanine Transaminase; AST: Aspartate Aminotransferase

Parameter	Unit	DAY 1	Reference Range
Haemoglobin	g/L	111	130–175
WBC	×10⁹/L	17.5	4.0–11.0
Platelets	×10⁹/L	89	150–400
INR	—	2.2	0.8–1.2
Bilirubin	µmol/L	180	5–21
ALT	U/L	71	7–56
AST	U/L	-	10–40
ALP	U/L	189	44–147
Albumin	g/L	13	35–50
Creatinine	µmol/L	174	60–110
Urea	mmol/L	17.2	2.5–7.5
Sodium	mmol/L	135	135–145
Potassium	mmol/L	4.4	3.5–5.0

A comprehensive liver screen to determine aetiology included viral hepatitis screening (hepatitis A, B, C, E - all negative), autoimmune markers (smooth muscle antibodies positive at 1:80 titre, but antinuclear antibody (ANA), liver-kidney microsomal (LKM), and antimitochondrial antibodies negative), and metabolic screening (normal alpha-1 antitrypsin, negative hemochromatosis gene analysis). Despite comprehensive investigation, the aetiology remained unknown.

Multimodal diagnostic evaluation

**Figure 1:**
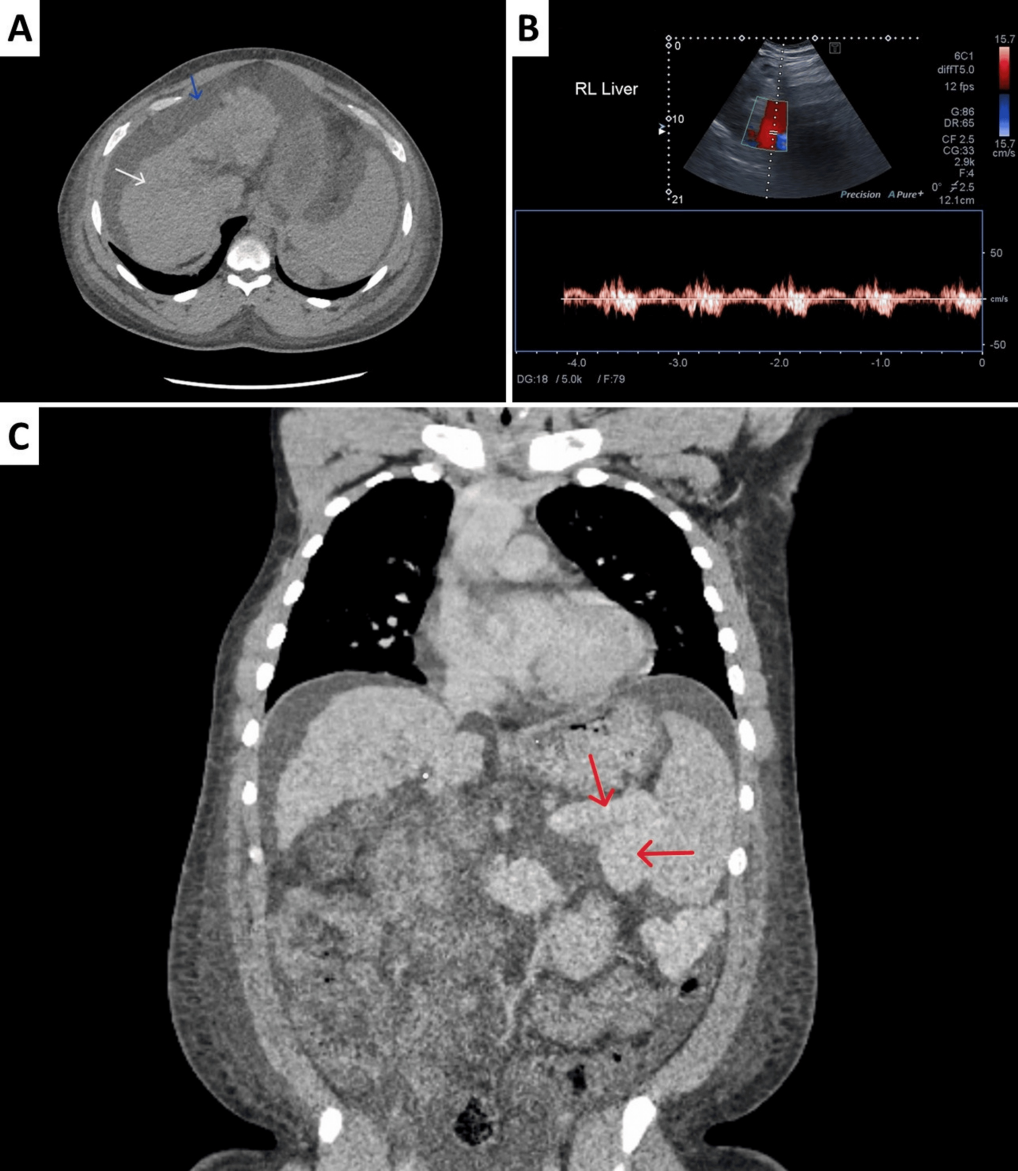
Imaging evaluation of liver cirrhosis and portosystemic shunting pre-transplant. A: CT liver triple-phase demonstrating cirrhotic morphology (white arrow) with moderate ascites (blue arrow) B: Doppler ultrasound of the portal vein showing a patent vessel with possible intermittent reverse flow, suggestive of portal hypertension. C: CT CAP with contrast showing tortuous dilatation of the splenic and left renal veins, consistent with a splenorenal shunt (red arrows) CT: computed tomography, CAP: chest-abdomen-pelvis

A computed tomography (CT) liver triple-phase showed a small, nodular cirrhotic liver with moderate ascites (Figure 1A). Abdominal ultrasound with portal vein Doppler demonstrated retrograde flow (Figure 1B), confirming severe portal hypertension. Notably, a CT chest-abdomen-pelvis with contrast, performed concurrently with the liver triple-phase scan, revealed a prominent, tortuous splenorenal shunt directly connecting the splenic hilum to the left renal vein (Figure 1C).

Additional findings included marked splenomegaly and a 2.5 cm benign pancreatic head cyst confirmed on magnetic resonance imaging (MRI). Importantly, no hepatocellular carcinoma or portal vein thrombosis was identified. 

**Figure 2:**
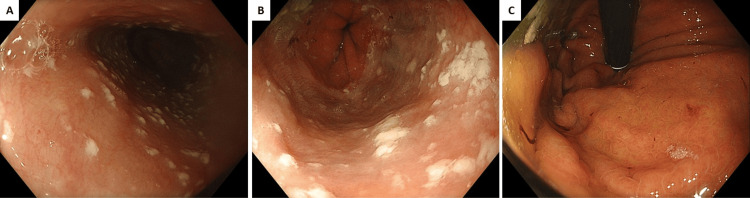
Upper gastrointestinal endoscopy showing absence of varices A: Middle oesophagus B: Lower oesophagus C: Stomach fundus

The clinical paradox became evident during upper gastrointestinal endoscopy performed for variceal surveillance. Despite clear radiological and clinical evidence of severe portal hypertension, endoscopy demonstrated complete absence of oesophageal or gastric varices (Figure 2), consistent with highly effective portal decompression via the extensive splenorenal shunt identified on cross-sectional imaging. 

**Figure 3:**
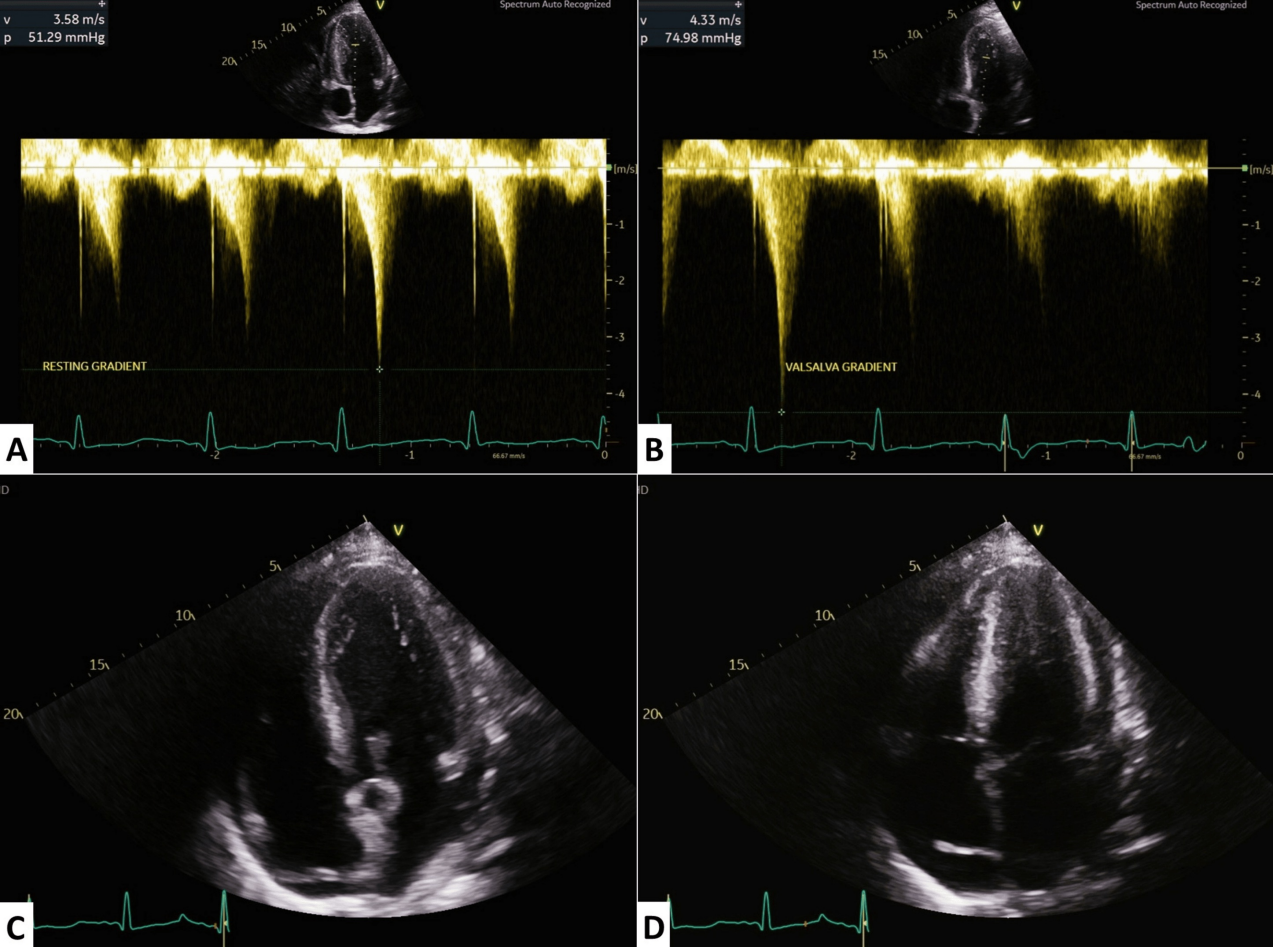
Transthoracic echocardiography demonstrating dynamic LVOT obstruction and hyperdynamic circulation. A: Continuous-wave Doppler across the LVOT at rest showing a gradient of 51.29 mmHg. B: Continuous-wave Doppler during the Valsalva manoeuvre demonstrating a marked increase in the LVOT gradient to 74.98 mmHg, confirming dynamic obstruction. C: Apical four-chamber view in diastole showing the LV cavity during filling. D: Apical four-chamber view in systole with near-complete LV cavity obliteration, highlighting the hypercontractile state. LV: left ventricle, LVOT: left ventricular outflow tract

Echocardiography revealed severe dynamic left ventricular outflow tract (LVOT) obstruction with a resting gradient of 51.29 mmHg (Figure 3A), dramatically increasing to 74.98 mmHg with Valsalva manoeuvre (Figure 3B), indicating hyperdynamic circulation secondary to portal hypertension (Figure 3C, 3D). Additional cardiac findings included concentric LV hypertrophy, hyperdynamic systolic function (ejection fraction (EF) >70%), impaired diastolic function (E/A ratio 0.8), and left atrial dilatation (48.3 mL/m²). 

Surgical intervention and postoperative course

Clinical deterioration with progressive organ failure necessitated urgent transplant listing. Pre-operative assessment identified the extensive splenorenal shunt as a significant operative risk requiring specialized surgical planning. 

Standard deceased donor liver transplantation proceeded with recipient hepatectomy and temporary portocaval shunting, followed by donor liver implantation with side-to-side cavocavostomy and portal vein anastomosis using 5-0 Prolene continuous sutures. Immediate visual assessment following portal reperfusion revealed distinct right posterior sector demarcation with colour change, providing unmistakable evidence of portal steal phenomenon where the low-resistance splenorenal shunt diverted critical portal flow away from the transplanted liver. Recognition of this life-threatening complication prompted immediate left renal vein ligation with placement of Surgical for haemostasis to eliminate the competing splenorenal pathway. Intraoperative ultrasound performed by the radiology team confirmed restored perfusion to all hepatic segments with normal waveforms, and visual inspection demonstrated complete resolution of hepatic sector demarcation, confirming adequate portal flow restoration to the graft. 

**Figure 4:**
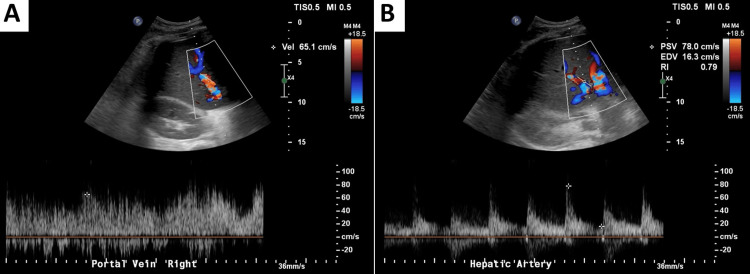
Post-transplant liver Doppler ultrasound A: Portal vein spectral Doppler demonstrating antegrade (hepatopetal) flow with a peak velocity of 65 cm/s, confirming adequate portal inflow to the graft. B: Hepatic artery spectral Doppler showing a peak systolic velocity of 78 cm/s with resistive index (RI) 0.79, consistent with satisfactory arterial perfusion.

Post-transplant imaging demonstrated successful graft placement with normal hepatic architecture and restored portal haemodynamics with antegrade flow (Figure 4).

Although initial graft function was satisfactory, transjugular liver biopsy on post-operative day five revealed moderate T-cell-mediated rejection (Banff grade 6/9), which was successfully managed with high-dose methylprednisolone pulse therapy. Explant histopathology revealed a cirrhotic specimen with nonspecific inflammation, confirming the cryptogenic nature of the underlying liver disease. The patient achieved complete recovery with sustained excellent graft function. At one-year follow-up, he remains asymptomatic with liver function tests within normal limits and triple-phase CT imaging demonstrating excellent graft perfusion with patent portal vein, normal hepatic arterial flow, and no evidence of collateral reformation or portal steal recurrence. 

## Discussion

Clinical paradox and the significance of absent varices 

This case illustrates a fundamental clinical paradox in which protective vascular adaptation can become a surgical challenge. The extensive splenorenal shunt, which had eliminated the risk of variceal bleeding even during the patient’s acute deterioration, subsequently threatened graft viability through a portal steal phenomenon [7], necessitating immediate surgical intervention. The development of such extensive collateral circulation in a patient of only 31 years is particularly remarkable, as extensive splenorenal shunts typically develop in the setting of advanced, longstanding portal hypertension. 

The absence of varices in established portal hypertension should not be misinterpreted as reassuring. Instead, it is a red flag that may indicate large-caliber alternative drainage pathways, such as splenorenal shunts, and warrants comprehensive evaluation. A well-performed contrast-enhanced CT, with optimal timing and administration, is essential to delineate vascular anatomy and identify potential flow-steal pathways before transplantation. Missing these on imaging risks intraoperative discovery, when graft perfusion is already compromised. Current clinical guidelines emphasize variceal surveillance and grading but provide limited guidance on interpreting their complete absence [8]. Targeted cross-sectional imaging is therefore critical to detect collaterals that, while appearing protective, may threaten surgical outcomes. 

Portal steal phenomenon and surgical management 

Portal steal phenomenon poses an immediate threat to graft survival when large-calibre portosystemic shunts, particularly splenorenal shunts exceeding 10 mm in diameter, provide a low-resistance pathway that preferentially diverts portal blood away from the transplanted liver [9]. 

Following restoration of portal inflow, visual demarcation of hepatic segments provides unmistakable intraoperative evidence of flow diversion, typically appearing as sharp colour differences between well-perfused and under-perfused regions [7]. Therefore, immediate recognition and correction are essential, as delayed intervention can compromise graft function. 

Left renal vein ligation represents a safe and effective surgical intervention for splenorenal steal, effectively eliminating the competing low-resistance pathway while preserving splenic and renal function [9]. This approach directly addresses the hemodynamic problem, unlike alternatives such as splenic artery embolization or splenectomy, and restores adequate portal perfusion, as confirmed by resolution of hepatic demarcation. This approach has been validated in multiple case series, with Tang et al. and Lee et al. demonstrating similar success rates for intraoperative and prophylactic left renal vein ligation respectively [10,11]. While endovascular management offers alternatives in select post-transplant cases [12], immediate surgical intervention remains essential when portal steal threatens graft viability intraoperatively. 

Implications for pre-transplant assessment and clinical practice 

This case highlights the importance of systematic pre-transplant evaluation of portal venous anatomy, particularly in patients with cryptogenic cirrhosis and extensive collateral development. Large shunts require careful surgical planning to prevent intraoperative flow diversion. Current guidelines emphasize variceal surveillance but provide limited guidance when varices are absent; cross-sectional imaging must specifically assess collateral networks that, while initially protective, may jeopardize transplant success. Thorough preoperative assessment and multidisciplinary planning are essential to transform potential hazards into manageable technical challenges, ensuring optimal graft outcomes [13]. 

## Conclusions

This case demonstrates that absent varices in severe portal hypertension signal extensive alternative drainage rather than mild disease - a critical distinction that determines transplant outcomes. The extensive splenorenal shunt that eliminated bleeding risk throughout this patient's acute illness became life-threatening during transplantation, causing portal steal that required immediate left renal vein ligation to prevent graft failure. The key clinical insight is that missing varices should trigger urgent cross-sectional imaging and specialized surgical planning, as the same anatomical adaptations that provide protection against bleeding create operative emergencies during liver transplantation. This paradox establishes absent varices as a diagnostic red flag requiring proactive vascular assessment rather than clinical reassurance.
